# Early Vascular and Morphological Response After Transvaginal Radiofrequency Ablation of Uterine Fibroids: A Doppler-Based Retrospective Study

**DOI:** 10.3390/jcm15135223

**Published:** 2026-07-03

**Authors:** Karolina Chmaj-Wierzchowska, Agnieszka Lach, Maja Bera, Klaudia Cieślicka, Filip Domagalski, Weronika Glaser, Zofia Kasprzak, Michalina Kowalczyk, Alan Bruszewski, Adam Malinger, Maciej Wilczak

**Affiliations:** 1 Department of Mother and Child Health and Minimally Invasive Gynecological Surgery, Poznan University of Medical Sciences, 60-701 Poznan, Poland; abruszewski@ump.edu.pl (A.B.); adam.malinger@ump.edu.pl (A.M.); mwil@ump.edu.pl (M.W.); 2 Laboratory of Modern Interventional Therapies in Gynecology and Urogynecology, Poznan University of Medical Sciences, 60-701 Poznan, Poland; 3 Faculty of Medicine, Poznan University of Medical Sciences, 60-701 Poznan, Poland; maja.bera01@gmail.com (M.B.); klaudia.cieslicka2207@gmail.com (K.C.); filip.domagalski2004@gmail.com (F.D.); glaserweronika@gmail.com (W.G.); zosia.kasprzak03@gmail.com (Z.K.); kmichalina57@gmail.com (M.K.)

**Keywords:** heavy menstrual bleeding, transvaginal radiofrequency ablation, Doppler ultrasound, vascularization, uterine fibroid

## Abstract

**Background/Objectives:** Uterine fibroids are one of the most prevalent forms of benign tumors in women and may substantially impair quality of life due to heavy menstrual bleeding, pelvic pain, and pressure-related symptoms. Transvaginal radiofrequency ablation (TV-RFA) has emerged as a promising minimally invasive, uterus-sparing treatment approach. However, there exists a paucity of data regarding the early vascular response evaluated through quantitative Doppler parameters. This study aimed to assess the short-term clinical outcomes and ultrasound effectiveness of TV-RFA in treating symptomatic uterine fibroids, with particular emphasis on early vascular and morphological response. **Methods:** This retrospective study included 38 women who presented with symptomatic uterine fibroids and underwent TV-RFA between July 2024 and December 2025. Inclusion criteria were as follows: (1) presence of up to three intramural fibroids (FIGO types 3–6) and (2) maximum diameter of fibroids: ≤6 cm. Patients were assessed at baseline and at 1- and 3-month follow-up visits. Ultrasound evaluation included the measurement of fibroid dimensions and volume as well as quantitative Doppler parameters (Pixels Power, Ratio, and CM^2^ Power Index). Clinical outcomes were assessed based on the intensity and duration of menstrual bleeding. Statistical analysis was performed using nonparametric tests with significance set at *p* < 0.05. **Results:** Significant reductions in fibroid dimensions and volume were observed at both follow-up time points, with the greatest effect at 3 months (*p* < 0.001). Doppler analysis demonstrated a marked decrease in vascularization parameters, particularly CM^2^ Power Index and Pixels Power (*p* < 0.001), suggesting an early vascular response to treatment. Clinically, the proportion of patients experiencing heavy menstrual bleeding considerably reduced, accompanied by a significant shortening of bleeding duration (*p* < 0.001). No major complications requiring surgical intervention were reported. **Conclusions:** TV-RFA was associated with significant short-term reductions in fibroid vascularization, fibroid volume, and bleeding-related symptoms in this cohort of women with symptomatic uterine fibroids. Quantitative Doppler parameters may serve as valuable early markers of treatment response; however, further studies with larger cohorts and a longer follow-up duration are warranted.

## 1. Introduction

Uterine fibroids represent one of the most common benign tumors of the female reproductive system. Their prevalence is influenced by various factors, including age, population, and the diagnostic method. Current research indicates that by the time women reach menopause, approximately 70–80% of women may experience fibroid occurrence, although only a fraction of these individuals develop clinical symptoms such as heavy menstrual bleeding, pelvic pain, or impaired fertility [1,2]. Treatment is primarily indicated for symptomatic patients or when the presence of fibroids considerably affects daily functioning. Therapeutic management includes both pharmacological and procedural approaches. Pharmacological treatment primarily aims to alleviate symptom severity, particularly abnormal uterine bleeding and pain, and in selected cases, to temporarily reduce fibroid volume [3,4,5,6,7,8,9,10,11]. However, medical therapy rarely facilitates permanent resolution of the disease and is mainly utilized as a bridging treatment or a preparatory measure for procedural intervention [12,13,14]. For many years, myomectomy and hysterectomy have remained the standard treatment options for symptomatic uterine fibroids.

In recent years, there has been a notable advancement in minimally invasive ablative techniques, including radiofrequency ablation (RFA) [15,16]. Transvaginal radiofrequency ablation (TV-RFA) is an ultrasound-guided procedure designed to induce controlled coagulative necrosis within the fibroid. Clinical data currently available indicate that TV-RFA may significantly reduce fibroid volume and alleviate symptoms, including bleeding and pain, in both short- and mid-term follow-up, with a relatively low rate of immediate complications and a short recovery period [17,18,19,20,21]. Reintervention rates also vary across studies depending on the applied technique, inclusion criteria, and lesion characteristics [22,23,24].

In contemporary uterine fibroid management, there has been increasing focus on personalized treatment strategies that consider patient preferences, reproductive plans, and the risk–benefit profile of individual therapeutic modalities [25]. Recent reports have shown that, in patients with focal adenomyosis, transcervical radiofrequency ablation may lead to lesion volume reduction, symptom improvement, and high patient satisfaction, with a favorable safety profile [26].

Appropriate patient selection is a crucial prerequisite for minimally invasive uterus-sparing therapies, including transvaginal radiofrequency ablation (TV-RFA). Although uterine leiomyomas are the most common benign tumors of the female reproductive tract, malignant mesenchymal tumors such as uterine sarcomas may occasionally present with clinical and imaging features overlapping those of benign fibroids. Consequently, accurate preprocedural imaging assessment is essential to minimize the risk of inappropriate treatment. Ultrasound remains the first-line imaging modality for the evaluation of myometrial lesions owing to its wide availability, low cost, and ability to assess lesion morphology and vascularization in real time. Previous studies have demonstrated that expert ultrasound examination can provide valuable information for differentiating benign and malignant uterine masses. Ludovisi et al. described the sonographic characteristics of uterine sarcomas and highlighted the role of ultrasound in identifying features suspicious for malignancy, while also reporting rare malignant lesions initially presenting as presumed benign uterine masses [27,28]. Furthermore, recent investigations have shown that structured clinical and ultrasound-based algorithms may improve the preoperative identification of mesenchymal uterine malignancies, including smooth muscle tumors of uncertain malignant potential (STUMP) [29]. In addition, ultrasound plays an important role in the diagnosis and follow-up of uncommon smooth muscle lesions, such as parasitic myomas, supporting individualized management strategies [30]. These findings emphasize the importance of comprehensive imaging evaluation before qualification for minimally invasive ablative procedures.

Despite the growing body of evidence supporting the efficacy of radiofrequency ablation, most available studies have focused predominantly on morphological outcomes, such as fibroid size and volume reduction. Data regarding early vascular response assessed using quantitative Doppler parameters remain limited. To our knowledge, few studies have systematically evaluated perfusion-related indices, such as CM^2^ Power Index or Pixels Power, as potential early biomarkers of treatment response following TV-RFA. Therefore, the present study aimed not only to assess morphological changes but also to investigate early vascular alterations and their temporal relationship with clinical and volumetric outcomes.

## 2. Materials and Methods

This retrospective study was conducted at the Ablation Clinic of the Department of Mother and Child Health and Minimally Invasive Gynecological Surgery, Poznan University of Medical Sciences. Thirty-eight patients who underwent TV-RFA of uterine fibroid(s) between 1 July 2024 and 31 December 2025 were included in the analysis. The inclusion criteria were as follows: (1) patients who were required to undergo TV-RFA due to uterine fibroid(s), (2) the maximum diameter of the largest fibroid did not exceed 6 cm prior to the procedure, and (3) patients who could complete full follow-up visits at 1 and 3 months after the procedure.

### 2.1. Inclusion and Exclusion Criteria for TV-RFA

Fibroids were classified according to the FIGO leiomyoma subclassification system. Patients exhibiting symptomatic intramural uterine fibroids were qualified to undergo TV-RFA. The eligibility criteria included the presence of up to three fibroids classified as FIGO types 3–6, with a maximum diameter not exceeding 6 cm. The exclusion criteria were as follows: (1) patients showing presence of pedunculated fibroids or fibroids of other locations corresponding to FIGO types 7–8; (2) patients suspected to have sarcoma or atypical fibroid on magnetic resonance imaging (MRI); (3) patients with gynecological malignancies; cervical dysplasia; infection of the vagina, cervix, or pelvis; or severe systemic diseases; and (4) patients who had planned future pregnancy. In patients presenting with heavy or abnormal uterine bleeding, malignant transformation was excluded based on endometrial and/or cervical canal biopsy performed under local anesthesia during hysteroscopy. Furthermore, to exclude atypical lesions, all patients underwent contrast-enhanced pelvic MRI within 6 months prior to the procedure. Because TV-RFA is a uterus-sparing ablative procedure and does not involve tissue excision, routine histopathological confirmation of treated fibroids was not available. Therefore, all patients underwent comprehensive preprocedural imaging assessment, including expert transvaginal ultrasound and contrast-enhanced pelvic MRI, to exclude lesions suspicious for malignancy.

### 2.2. Pre-TVRFA Ultrasound Examination

Prior to TV-RFA of uterine fibroids, a thorough ultrasound examination was conducted to assess the number, dimensions, and location of fibroids. Each lesion was classified according to the FIGO system into one of the following four categories: submucosal, intramural, subserosal, or mixed type.

The mean diameter of each fibroid was calculated using the following formula:

Mean diameter = (length + width + height)/3

Fibroid volume, which was subsequently used for analyzing volume reduction, was consistently calculated using the standard ellipsoid formula:

Volume = (4/3) × π × (length/2 × width/2 × depth/2)

Fibroid vascularization was quantitatively assessed based on the Doppler signal within a region of interest (ROI). The following parameters were analyzed: Pixels Power, Pixels ROI, Ratio, CM^2^ Power Index, and CM^2^ ROI. Pixels Power indicates the total intensity of the vascular signal within the lesion, Pixels ROI denotes the size of the analyzed area expressed in pixels, and the Ratio represents the relationship between signal intensity and ROI size. Additionally, we analyzed the square-centimeter power index (CM^2^ Power Index), which is defined as the vascular signal intensity normalized per unit area, while CM^2^ ROI corresponds to the area of interest expressed in cm^2^. These parameters were used to quantitatively assess fibroid perfusion before treatment and during follow-up examinations after the procedure. All ultrasound examinations were performed by the same expert operators certified in gynecological ultrasound, each with extensive experience (>10 years) in the assessment of uterine pathology, using the same ultrasound system and identical imaging settings to minimize inter-operator and inter-instrument variability.

### 2.3. Description of the RFA System

A radiofrequency ablation generator, the VIVA RF System (STARmed Co., Ltd., Goyang, Republic of Korea), was used for the ablation procedure. The system was equipped with a fixed RF coagulation electrode, 17G, 35 cm length, with a 1 cm active tip (STARmed Co., Ltd.; reference number: 17–35s30F). The system was cooled using continuous infusion delivered through an infusion pump. The generator operated at 480 kHz frequency, enabling the maintenance of the electrode tip temperature within the range of 60–90 °C. This elevated temperature induced protein denaturation and formation of coagulative necrosis within the fibroid. Although the maximum generator output was 200 W, a power setting of 35 W was used in all procedures in our study.

### 2.4. Description of the TV-RFA Procedure

TV-RFA was performed under general anesthesia and ultrasound guidance. The needle electrode was introduced through the anterior or posterior vaginal fornix into the fibroid, ensuring that the tip was positioned approximately 0.5 cm from the pseudocapsule of the lesion. Throughout the application process, output power and changes in tissue impedance were continuously monitored on the generator display. The effectiveness of ablation in the specified area was confirmed ultrasonographically by the persistent change in echogenicity of the treated tissue, manifested as hyperechogenicity. Subsequently, the electrode was repositioned into adjacent, previously untreated portions of the fibroid, and ablation was continued until the echogenic changes encompassed approximately 80% of the lesion volume. Following completion of the procedure, the presence of vaginal and cervical bleeding was assessed, and bladder catheterization was performed.

### 2.5. Follow-Up at 1 and 3 Months After TV-RFA

Patients underwent routine evaluation at 1 and 3 months post-procedure to assess the safety and efficacy of TV-RFA. The follow-up visit included a clinical interview with a specific focus on changes in the severity of menstrual bleeding, including its volume and duration. Transvaginal ultrasound (TVUS) was performed to measure the maximum dimensions of the target fibroid(s) and calculate their volume (cm^3^) using a standardized measurement methodology. Fibroid vascularization was assessed by Doppler ultrasound, accompanied by an examination of intralesional necrotic features. We also evaluated whether necrotic areas encompassed at least 80–90% of the lesion volume on imaging. During each clinical visit, reported symptoms, any complications encountered, need for additional interventions, and instances of hospitalization were recorded. When necessary, adverse events were classified, and further management decisions were made, including urgent additional diagnostic work-up, pharmacological treatment, or specialist consultation.

Adverse events were classified according to the Clavien–Dindo Classification of Surgical Complications, in which Grade I–II complications require only pharmacological or minor interventions, Grade III complications require surgical, endoscopic, or radiological intervention, Grade IV complications are life-threatening, and Grade V corresponds to death.

### 2.6. Assessment of Procedure Efficacy

The efficacy of the procedure was evaluated by comparing the linear dimensions of the fibroid (length, width, and depth) and its volume between baseline and follow-up visits at 1 and 3 months. The relative volume reduction of the lesion was also calculated. Additionally, changes in vascularization parameters, including CM^2^ Power Index, Pixels Power, Ratio, and ROI area, were analyzed, together with their relationship with patients’ anthropometric characteristics and procedure duration. All imaging measurements and clinical data were recorded in the study case report forms. The primary endpoints were established as the absolute and relative change in fibroid volume and the change in clinical bleeding pattern at 3 months post-procedure. Safety was assessed by monitoring the incidence of complications and the necessity for reinterventions during the 3-month follow-up period. All Doppler settings (gain, pulse repetition frequency, wall filter) were kept constant across examinations. All imaging examinations were conducted by operators trained in gynecological ultrasound, following a standardized measurement protocol.

### 2.7. GenAI

During the preparation of this manuscript/study, the author(s) used [ChatGPT (OpenAI, GPT-5.3, accessed April 2026)] for the purposes of language editing and improvement of readability, paraphrasing and refining the wording of the manuscript. The authors have reviewed and edited the output and take full responsibility for the content of this publication.

### 2.8. Statistical Analysis

Data analysis was performed using Statistica (Cloud Software Group, Inc. Fort Lauderdale, FL, USA (2023), Data Science Workbench, version 14), The Jamovi Project (2022), Jamovi (Version 2.3) [Computer Software, Sydney, NSW, Australia], and Microsoft Excel (Microsoft Office, Redmond, WA, USA (2019), version 2205). The normality of variable distribution was assessed using the Shapiro–Wilk test. Comparisons across three time points were performed using the Friedman test, a non-parametric equivalent to repeated-measures ANOVA, with post hoc pairwise comparisons using the Durbin–Conover test. Technical ultrasound indices were compared using the Wilcoxon signed-rank test. Effect size was assessed using the rank-biserial correlation coefficient. For the nominal variable of menstrual bleeding severity, comparisons across the three time points were performed with Cochran’s Q test, with pairwise comparisons using McNemar’s chi-square test. Effect size was assessed using the Phi coefficient. Relationships between quantitative variables were analyzed using Spearman’s rank correlation coefficient. A *p*-value of <0.05 was considered statistically significant for all analyses.

## 3. Results

### 3.1. Characteristics of the Study Group

The age distribution of the study group revealed that patients were in the late reproductive and perimenopausal period, which corresponds to the typical clinical window for uterine fibroid development. An analysis of anthropometric parameters showed that the mean BMI was at the threshold of overweight classification. Table 1 presents the anthropometric measurements of the study population.

Patient distribution according to the Funaki classification showed an almost equal representation of types I and II. The prevalence of intramural fibroids reflected the most frequently observed clinical scenario encountered in gynecological practice. Several patients reported a history of previous surgical procedures and associated comorbidities. The majority of the cohort was not undergoing hormonal treatment. Table 2 presents the characteristics of the study population.

Fertility-related data presented in Table 3 describe a cohort consisting of women who had largely completed their reproductive plans, although the presence of nulliparous patients underscores the importance of uterus-sparing treatment options; however, fertility outcomes were beyond the scope of the present study because women planning future pregnancy were excluded. Notably, no deliveries by cesarean section were reported in the study population, which is a favorable outcome for the technical aspects of the procedure as it eliminates concerns related to uterine scarring. The observation of a stable pattern of vaginal deliveries and a low rate of prior miscarriages indicates that the cohort had a relatively healthy obstetric history prior to the development of fibroid pathology.

The duration of the procedure ranged from 10 to 30 min. The mean procedure time was 17.4 ± 4.8 min (95% CI: 15.79–19.0), and the median was 15 min.

### 3.2. Vascularization

We observed a highly significant reduction in most hemodynamic parameters. The magnitude of this effect was assessed as very large for the CM^2^ Power Index and large for the Pixels Power Index and the Ratio parameter. The ROI area (Pixels) alone showed no significant changes, indicating the absence of correlation between the procedure and the total area of the measurement field (Table 4).

The analysis of correlations identified limited significant relationships. A moderate negative correlation was observed between procedure duration and the post-procedural CM^2^ Power Index, while a weak positive correlation was found between body weight and the vascularized area. Patient age was not significantly associated with any of the technical parameters of the procedure (Table 5).

### 3.3. Ultrasound Analysis

Changes in lesion volume measured by ultrasound before treatment and at 1- and 3-month follow-up. Values are expressed as mean, standard deviation, median, and range. The lesion volume progressively reduced over time.

The mean lesion volume decreased from 66.2 before treatment to 41.0 at 1 month and to 26.1 at 3 months (Table 6). This corresponds to a mean reduction of 38% at 1 month and 60% at 3 months. The observed volume reduction was statistically significant at both 1 month and 3 months (*p* < 0.001).

**Figure 1:**
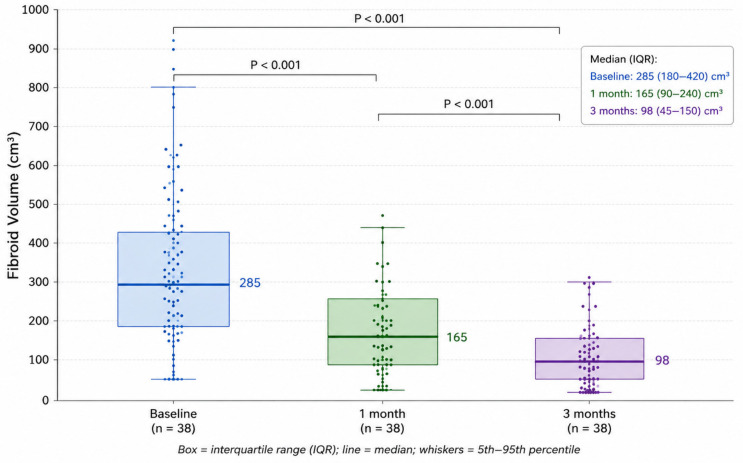
Changes in fibroid volume before treatment and during follow-up after transvaginal radiofrequency ablation (TV-RFA). Box-and-whisker plots illustrating fibroid volume at baseline and at 1- and 3-month follow-up visits. The boxes represent the interquartile range (IQR), the horizontal line within each box indicates the median, and the whiskers represent the 5th–95th percentiles. A progressive reduction in fibroid volume was observed over time, with the greatest decrease at 3 months after treatment (*p* < 0.001).

The observed volume reduction was statistically significant at both 1 month and 3 months (*p* < 0.001) (Figure 1).

Ultrasound analysis showed significant changes in the linear dimensions of fibroids over time (Table 7). Pairwise comparisons confirmed a significant reduction in fibroid length at 1 month after the procedure, with a further increase in effect observed at 3 months (Friedman ANOVA: χ^2^ = 28.2; df = 2; *p* < 0.001). A significant reduction in fibroid width was confirmed at the final follow-up point, while the change in fibroid width at 1 month reached a borderline level of significance (Friedman ANOVA: χ^2^ = 31.2; df = 2; *p* < 0.001). The most prominent changes were observed in the third dimension (fibroid depth), where a significant reduction was evident already at 1 month and persisted throughout the follow-up period (Friedman ANOVA: χ^2^ = 45.2; df = 2; *p* < 0.001). Fibroid volume demonstrated a continuous, progressive decrease across time points, with the greatest reduction observed at 3 months (Friedman ANOVA: χ^2^ = 41.7; df = 2; *p* < 0.001).

We found significant negative correlations between body weight and BMI and fibroid dimensions at postoperative follow-up (Table 8). The strength of these correlations ranged from moderate to strong, suggesting that anthropometric parameters are influenced by the dynamics of dimensional changes. Procedure duration did not significantly impact ultrasound-measured fibroid dimensions.

### 3.4. Menstrual Bleeding

The treatment had a significant effect on the severity of menstrual bleeding (Q = 35.83; *p* < 0.001). At the first follow-up, a 75% effectiveness in reducing heavy menstrual bleeding was observed, with a significant difference and a moderate effect size (χ^2^ = 16.06; *p* < 0.001; ϕ = 0.33). Between baseline and the 3-month follow-up, the effectiveness in reducing menstrual bleeding reached 92%, with a significant difference and a weak association effect (χ^2^ = 20.05; *p* < 0.001; ϕ = 0.18). The weak effect size of the Phi coefficient is most likely attributable to the marked asymmetry of the distribution, which reduces its value in the analysis of this variable. Furthermore, based on the proportions before and after treatment [(32/6)/(14/24)], the odds ratio calculated for the 1-month follow-up indicated that the odds of absence of heavy menstrual bleeding were more than 9-fold higher 1 month post-procedure compared to that at baseline. For the 3-month follow-up [(36/2)/(14/24)], the odds were almost 31-fold higher than before treatment. The treatment effect showed a nonsignificant difference between the 1- and 3-month follow-up (χ^2^ = 1.5; *p* = 0.22; ϕ = 0.22). However, it is noteworthy that one case of recurrent heavy menstrual bleeding was observed at 3 months post-treatment.

The findings revealed a significant reduction in menstrual duration (Table 9). The effect size compared to baseline was maximal (complete association) at both follow-up time points. The absence of significant differences between the 1- and 3-month follow-ups indicates that the reduction in bleeding duration stabilizes immediately after the procedure.

The analysis showed no significant associations between menstrual duration and anthropometric measurements or procedure duration (Table 10). A moderate negative correlation between body weight and bleeding duration was observed only at the 3-month follow-up.

### 3.5. Complications

None of the patients experienced any complications according to the Clavien–Dindo Classification of Surgical Complications. However, given the limited sample size and a short follow-up duration, we cannot fully exclude rare complications.

## 4. Discussion

Uterine fibroids are one of the most common benign uterine lesions and can considerably impair patients’ quality of life due to heavy menstrual bleeding, pain, and pressure-related symptoms. While traditional surgical approaches are highly effective, they are associated with greater invasiveness and longer recovery time. TV-RFA represents a modern, minimally invasive uterus-sparing treatment modality. Imaging assessment revealed a notable and progressive reduction in both linear dimensions and volume of fibroids across successive follow-up time points.

The reduction in fibroid length was already significant at 1 month, while the effect became even more pronounced at the 3-month follow-up. Unlike conventional approaches based primarily on morphological evaluation, this study demonstrates that vascular changes occur earlier and may represent a more sensitive indicator of the initial therapeutic effect. Importantly, the identification of a biphasic response pattern—early vascular suppression followed by delayed morphological regression—provides novel insight into the temporal dynamics of fibroid involution after TV-RFA.

In contrast to most existing studies, in which treatment efficacy was primarily assessed based on morphological parameters, the present study employed quantitative perfusion analysis using Doppler-derived indices. A noteworthy finding of this study is the significant reduction in Doppler parameters, particularly the CM^2^ Power Index, Pixels Power, and Ratio, whereas the ROI area expressed in pixels did not alter significantly. Although the observed reductions in CM^2^ Power Index, Pixels Power, and related Doppler-derived parameters suggest that vascular changes may occur earlier than morphological regression, these findings should be interpreted cautiously. The reproducibility of these measurements was not assessed in the present study, and validated clinical thresholds are currently lacking. Therefore, these parameters should presently be regarded as exploratory imaging markers requiring further prospective validation before they can be incorporated into routine clinical decision-making.

Unlike conventional analyses based solely on morphological changes, the present study performed quantitative perfusion assessment for detecting an early tissue response to treatment. The results indicate that the earliest detectable effect of treatment is a reduction in fibroid perfusion, followed by morphological involution. This interpretation is consistent with the mechanism of coagulative necrosis reported for radiofrequency ablation techniques [15,16,24,31]. In this context, vascular parameters, particularly the CM^2^ Power Index, may serve as valuable markers of early treatment response, although their prognostic value requires further confirmation in studies with longer follow-up periods. The observed pattern of changes—rapid reduction in vascularization followed by a decrease in fibroid dimensions and volume—is biologically plausible and corresponds to the mechanism of action of RFA. Radiofrequency energy leads to tissue heating, protein denaturation, and the formation of coagulative necrosis, which over subsequent weeks and months promotes remodeling of the treated lesion [15].

In this context, our results support the concept that vascular changes may precede a complete morphological response. It is important to note that very few studies on uterine fibroid treatment have applied quantitative Doppler indices such as the CM^2^ Power Index and Pixels Power. Therefore, the current findings may constitute an important contribution to the development of methods for monitoring the effects of ablative therapies. However, the prospective utilization of these parameters as markers of early treatment response requires further exploration, particularly for their prognostic value and correlation with long-term fibroid volume reduction and clinical improvement.

Another key finding of our present study was the clinical improvement in menstrual bleeding patterns. At 1 month after the procedure, the proportion of patients reporting heavy menstrual bleeding substantially reduced; this effect was maintained at the 3-month follow-up. A significant reduction in menstrual bleeding duration was also observed, which was evident in the early postoperative period. These observations are consistent with published data indicating that RFA techniques may effectively alleviate symptoms associated with uterine fibroids, particularly heavy menstrual bleeding and symptoms related to the presence of the lesion [2,13,16,24,31].

Observational data from studies conducted by Santalla-Hernández et al. [17,18,19,20] also confirmed the high efficacy of this treatment modality, with an average fibroid size reduction of 40–60% within 6 months of treatment. The commonly noted benefits include a reduction in menstruation duration, a decrease in bleeding severity by more than 50%, and a high level of patient satisfaction [17,18,19,20]. It should, however, be noted that the present study utilized patient-reported history to assess clinical symptoms, without using standardized quantitative tools for evaluating bleeding severity; this aspect limits direct comparability with some prospective studies.

In the present analysis, selected associations between anthropometric parameters and treatment response were also observed. Body weight and BMI were moderately negatively correlated with selected fibroid dimensional parameters in follow-up examinations; additionally, isolated associations were noted between procedure duration and post-treatment vascularization parameters. However, these findings should be interpreted with caution. On the one hand, these observations may reflect true biological differences related to the influence of body weight and the hormonal–inflammatory milieu on the pathophysiology of uterine fibroids [2,32]. On the other hand, they may result from the relatively small cohort size and the exploratory nature of the analysis.

In the multicenter prospective study by Santalla-Hernández et al. [21], smaller baseline fibroid volume and patient age ≥ 41 years were identified as independent predictors of greater reduction in uterine fibroid volume. A critical finding is that the mean procedure duration was short, which confirms the practical and minimally invasive nature of TV-RFA. This is a crucial feature from a clinical perspective, as contemporary management of uterine fibroids is increasingly shifting to uterus-sparing approaches that reduce perioperative trauma, shorten recovery time, and better align therapy with patient expectations [21,31]. The results of our current study are consistent with this trend and suggest that TV-RFA may represent a valuable treatment option for appropriately selected patients with symptomatic uterine fibroids.

Our research findings align closely with the available data on the efficacy of RFA in treating uterine fibroids. Systematic reviews and meta-analyses have demonstrated that RF-based ablative procedures can reduce fibroid volume and alleviate symptoms, with a relatively short recovery time and high levels of patient satisfaction [16,17,18,19,20,21]. It should also be noted that results across individual studies may vary depending on the applied technique, lesion access, inclusion criteria, fibroid size and location, and follow-up duration [16].

Although both our observations and the available literature suggest a favorable clinical profile of RFA techniques, including TV-RFA, in longer follow-up [16,17,18,19,20,21], the precise position of this procedure within treatment algorithms requires further comparative studies. Nevertheless, the short procedure time, no requirement for hospitalization, and documented ultrasound-based efficacy make radiofrequency ablation a promising minimally invasive alternative to more invasive surgical treatment in appropriately selected patients. TV-RFA shows a distinct biphasic response characterized by early vascular suppression followed by progressive morphological regression of uterine fibroids. Quantitative Doppler parameters, particularly the CM^2^ Power Index, appear to be promising early biomarkers of treatment response, potentially enabling more precise and earlier monitoring of therapeutic efficacy. Although these findings support the inclusion of vascular assessment in post-procedural evaluation protocols, prospective validation in larger cohorts is required. In clinical practice, these findings may support a two-step follow-up strategy, with early Doppler assessment to evaluate immediate treatment response, followed by volumetric assessment at later time points.

Recent advances in radiomics, artificial intelligence, and machine learning have opened new perspectives in the diagnostic assessment of uterine mesenchymal tumors. Several studies have demonstrated that quantitative imaging features extracted from ultrasound and magnetic resonance imaging may improve the differentiation between benign leiomyomas and uterine sarcomas. Zheng et al. [33] reported promising results using radiomics-based approaches for distinguishing uterine leiomyomas from sarcomas, highlighting the potential of advanced image analysis to support clinical decision-making [33]. Similarly, the AROMA pilot study demonstrated the feasibility of radiomic models in gynecologic oncology and their ability to identify patients with ovarian malignancies [34]. More recently, Ciccarone et al. [35] developed a radiomics-based model incorporating imaging features and patient age, which achieved encouraging diagnostic performance in differentiating uterine sarcomas from leiomyomas [35]. Although the present study focused on treatment response assessment rather than diagnostic classification, these developments suggest that future integration of quantitative Doppler parameters with radiomic and machine-learning approaches may further improve patient selection, treatment monitoring, and personalized management strategies for women with uterine fibroids [36].

### 4.1. Clinical Implications

The findings of this study may have direct implications for the clinical management and follow-up of patients undergoing transvaginal radiofrequency ablation (TV-RFA) for uterine fibroids. The observed early and significant reduction in Doppler-derived vascularization parameters, particularly the CM^2^ Power Index, suggests that vascular changes may serve as sensitive early indicators of treatment response, preceding measurable morphological regression.

From a practical perspective, this may support the incorporation of quantitative Doppler assessment into early post-procedural follow-up protocols. In contrast to fibroid volume, which demonstrates a gradual reduction over time, vascular parameters appear to reflect the immediate biological effect of ablation, namely perfusion shutdown and the initiation of coagulative necrosis.

Consequently, Doppler evaluation could potentially enable earlier identification of treatment efficacy or failure, allowing for more timely clinical decision-making. This may be particularly relevant in patients with persistent symptoms or in cases where early assessment of treatment success is clinically desirable.

While volumetric assessment remains the standard for monitoring long-term outcomes, the present findings suggest that Doppler-based indices may complement, and in selected cases partially substitute, early morphological evaluation. However, further prospective studies are required to establish standardized thresholds and validate the prognostic value of these parameters in routine clinical practice.

### 4.2. Limitations of the Study

The main limitations of this study include the small sample size, single-center design, lack of a control group, and a short 3-month follow-up period. These limitations restrict the ability to assess the durability of the effect, the risk of symptom recurrence, the rate of subsequent interventions, and the impact of the procedure on reproductive outcomes. Additionally, ultrasound assessment, particularly Doppler parameters, remains partially operator-dependent despite the use of a standardized measurement protocol. Hence, the obtained results should be interpreted as promising but preliminary observations. Furthermore, the 3-month follow-up period was specifically designed to assess early vascular and morphological changes after TV-RFA and does not permit evaluation of long-term treatment durability, recurrence rates, reproductive outcomes, or delayed adverse events. An additional limitation of the present study is that symptom improvement was assessed based on patient-reported clinical history rather than validated instruments designed to quantify menstrual bleeding and fibroid-related symptom burden. Recent studies have emphasized the importance of incorporating standardized patient-reported outcome measures and quality-of-life assessments when evaluating treatment effectiveness in women with uterine fibroids [37]. Therefore, future studies should combine objective imaging findings with validated symptom and quality-of-life questionnaires to provide a more comprehensive evaluation of treatment outcomes. Potential selection and attrition bias should also be considered, as only patients who completed both follow-up visits were included in the final analysis. Consequently, individuals with persistent symptoms, complications, or less favorable treatment responses may have been underrepresented, potentially influencing the observed outcomes and limiting the generalizability of the findings. Although no major complications were observed during the study period, the relatively small sample size and short follow-up duration limit the ability to identify uncommon adverse events and delayed complications. Therefore, the present findings should not be interpreted as definitive evidence of procedural safety, and further studies with larger cohorts and longer follow-up are required to more comprehensively evaluate the safety profile of TV-RFA.

### 4.3. Strengths of the Study

Several strengths of the present study should be highlighted. First, unlike most previous studies on transvaginal radiofrequency ablation (TV-RFA), which primarily focused on morphological outcomes, our analysis incorporated quantitative Doppler-derived vascularization parameters, including the CM^2^ Power Index and Pixels Power. This approach enabled a more comprehensive assessment of the biological response to treatment and provided insight into early perfusion changes occurring after ablation.

Second, ultrasound examinations were performed using the same ultrasound system, identical imaging settings, and a standardized measurement protocol, which minimized technical variability and enhanced the reliability and reproducibility of the obtained results.

Third, the study simultaneously evaluated both vascular and morphological responses, allowing characterization of the temporal sequence of treatment effects. This combined assessment demonstrated that vascular suppression precedes measurable fibroid shrinkage, providing novel information regarding the mechanism and dynamics of tissue involution following TV-RFA.

Another important strength is the inclusion of clinically meaningful outcomes, such as changes in menstrual bleeding severity and duration, which directly reflect treatment effectiveness from the patient’s perspective and complement imaging-based findings.

Finally, the study was conducted in a real-world clinical setting and reflects routine practice in a tertiary referral center. Therefore, the findings may have practical relevance for everyday clinical management of patients undergoing TV-RFA for symptomatic uterine fibroids.

## 5. Conclusions

In this retrospective cohort, TV-RFA was associated with favorable short-term vascular, morphological, and clinical outcomes. However, these findings should be interpreted cautiously because of the limited sample size, absence of a control group, and short follow-up duration. The obtained results indicate a biphasic treatment response, characterized by early and pronounced suppression of fibroid vascularization followed by progressive morphological regression. Quantitative Doppler parameters, particularly the CM^2^ Power Index, may represent promising early biomarkers of treatment efficacy, enabling more precise monitoring of therapeutic response compared to conventional morphological assessment. The inclusion of vascular assessment in standard follow-up after TV-RFA may potentially increase the sensitivity of detecting treatment response. Because of limitations of the current study, including its retrospective design, small sample size, and short follow-up period, the presented results require confirmation in prospective studies with larger cohorts and longer follow-up.

## Figures and Tables

**Table 1:** Anthropometric measurements.

	M ± SD	Min–Max	Me [Q1–Q3]	95% CI
Age	44.3 ± 5.3	32–53	45.5 [41–49]	42.58–46.1
Height (cm)	166.9 ± 6.2	153–178	168 [161–172]	164.82–168.9
Body weight (kg)	71.3 ± 15.6	48–100	68.5 [55–82]	66.2–76.4
BMI	25.5 ± 4.8	18.38–35.7	24.89 [21.48–28.7]	23.9–27.1

**Table 2:** Characteristics of the study population.

		N	%
Funaki Classification	Type I	18	47.37%
	Type II	20	52.63%
Type	Intramural	34	89.47%
	Submucosal	2	5.26%
	Subserosal	2	5.26%
Surgical history	Yes	20	52.63%
	No	18	47.37%
Comorbidities	Yes	21	55.26%
	No	17	44.74%
Anemia	Yes	6	15.79%
	No	32	84.21%
Hormonal therapy	Yes	11	28.95%
	No	27	71.05%
Body mass assessment	Underweight	1	2.63%
	Normal	18	47.37%
	Overweight	11	28.95%
	Obesity	8	21.05%

**Table 3:** Obstetric history.

		N	%
Number of deliveries	0	15	39.47%
	1	9	23.68%
	2	10	26.32%
	3	3	7.89%
	4	1	2.63%
Number of miscarriages	0	31	81.58%
	1	1	2.63%
	2	1	2.63%
	3	5	13.16%
Vaginal delivery	0	16	42.11%
	1	11	28.95%
	2	8	21.05%
	3	2	5.26%
	4	1	2.63%
Cesarean section	0	38	100.00%

**Table 4:** Technical parameters of vascularization.

		M ± SD	Min–Max	Me [Q1–Q3]	W	*p*	Rs
INDEX PIXELS POWER	Before	25,869.5 ± 60,367.5	2957–377,547	10,872.5 [8412–22,523]	632	<0.001	0.71
	After	9429.3 ± 10,969.9	864–50,394	6587.5 [2732–7459]			
PIXELS ROI	Before	118,060.3 ± 155,502	13,567–990,909	77,434.5 [45,860–161,680]	437	0.34	0.18
	After	91,142.3 ± 31,172.5	48,178–166,222	78,269 [71,680–118,538]			
RATIO	Before	16.1 ± 11.3	2.78–46.5	12.3 [6.6–26.8]	622	<0.001	0.68
	After	8.5 ± 7.3	1.5–39	6.05 [3.7–12.8]			
INDEX CM^2^ POWER	Before	4 ± 4.2	0.58–20	3.53 [1.19–5.1]	670	<0.001	0.81
	After	2.2 ± 2.2	0.1–10	1.98 [0.62–2.4]			
CM^2^ ROI	Before	26.9 ± 24.4	3.66–88.3	17.7 [13.62–38.7]	610	<0.001	0.65
	After	18.9 ± 11.9	3.9–45.9	15.52 [10.07–21.3]			

**Table 5:** Spearman’s rank correlation coefficient (R) between technical vascularization parameters, anthropometric measurements, and procedure duration.

	Age		Body Weight		BMI		Procedure Duration	
	Before	After	Before	After	Before	After	Before	After
INDEX PIXELS POWER	−0.22	0.08	−0.07	0.24	−0.09	0.18	0.21	−0.29
PIXELS ROI	−0.22	−0.21	0.18	0.35 *	0.13	0.26	−0.18	−0.14
RATIO	−0.25	0.07	0.11	0.05	0.15	0.06	0.18	−0.31
INDEX CM^2^ POWER	−0.23	−0.03	0.33 *	0.13	0.24	0.04	0.22	−0.38 *
CM^2^ ROI	−0.07	−0.12	0.22	0.25	0.16	0.22	−0.09	−0.2

* *p* < 0.05.

**Table 6:** Changes in lesion volume over time (n = 38).

Parameter	Baseline (Pretreatment)	1 Month	3 Months
Mean volume	66.20	41.01	26.05
Standard deviation (SD)	109.43	49.87	33.92
Median	36.32	31.59	21.33
Minimum	4.17	1.53	1.00
Maximum	572.30	194.90	123.24
Mean change vs. baseline	—	−25.19	−40.15
Mean % reduction	—	38%	60%

**Table 7:** Assessment of uterine fibroid dimensions by ultrasound examination.

Length	M ± SD	Min–Max	Me [Q1–Q3]	95% CI	D-C	*p*	Rs
A	4.3 ± 1.5	2–8.9	4.3 [3.1–5.3]	3.82–4.8	2.61 ^ab^	0.01	0.36
B	4.1 ± 1.5	1.6–7.4	4.15 [3–5.4]	3.64–4.6	6.57 ^ac^	<0.001	0.70
C	3.7 ± 1.4	1–6.5	3.5 [2.5–5]	3.19–4.1	3.96 ^bc^	<0.001	0.85
**Width**	**M ± SD**	**Min–Max**	**Me [Q1–Q3]**	**95% CI**	**D-C**	***p***	**Rs**
A	4.3 ± 2	2–11.5	4.1 [3–4.7]	3.65–5	1.99 ^ab^	0.05	0.50
B	3.8 ± 1.4	1.4–6.7	3.75 [2.8–4.5]	3.34–4.3	6.96 ^ac^	<0.001	0.80
C	3.2 ± 1.2	1.08–5.5	3.2 [2.41–4.2]	2.82–3.6	4.97 ^bc^	<0.001	0.94
**Depth (third dimension)**	**M ± SD**	**Min–Max**	**Me [Q1–Q3]**	**95% CI**	**D-C**	***p***	**Rs**
A	4.4 ± 1.8	2–10.7	4.05 [3–5]	3.78–5	5.18 ^ab^	<0.001	0.72
B	3.7 ± 1.4	1.2–7.5	3.5 [2.7–4.5]	3.28–4.2	10.63 ^ac^	<0.001	0.91
C	3.1 ± 1.3	1.2–6.5	3 [2–4.2]	2.71–3.6	5.45 ^bc^	<0.001	0.91
**Volume**	**M ± SD**	**Min–Max**	**Me [Q1–Q3]**	**95% CI**	**D-C**	***p***	**Rs**
A	66.2 ± 104.9	4.17–572.3	38.15 [16.04–67.2]	31.74–100.7	2.87 ^ab^	0.005	0.53
B	41 ± 39	1.53–194.9	33.16 [11.39–57.1]	28.19–53.8	9.27 ^ac^	<0.001	0.84
C	26.1 ± 25.3	1–123.2	21.31 [5.87–42.6]	17.74–34.4	6.41 ^bc^	<0.001	0.92

Length—Friedman ANOVA: χ^2^ = 28.2; df = 2; *p* < 0.001; Width—Friedman ANOVA: χ^2^ = 31.2; df = 2; *p* < 0.001; Depth (third dimension)—Friedman ANOVA: χ^2^ = 45.2; df = 2; *p* < 0.001; Volume—Friedman ANOVA: χ^2^ = 41.7; df = 2; *p* < 0.001. Post hoc pairwise comparisons were performed using the Durbin–Conover test (D-C). Superscript letters indicate statistically significant pairwise comparisons: ^ab^ = comparison between baseline (A) and 1-month follow-up (B); ^ac^ = comparison between baseline (A) and 3-month follow-up (C); ^bc^ = comparison between 1-month (B) and 3-month follow-up (C).

**Table 8:** Spearman’s rank correlation coefficient (R) between ultrasound measurements, anthropometric parameters, and procedure duration.

	Age			Body Weight			BMI			Procedure Duration		
	Before	1 mo.	3 mo.	Before	1 mo.	3 mo.	Before	1 mo.	3 mo.	Before	1 mo.	3 mo.
I	0.25	0.26	0.26	−0.25	−0.4 *	−0.35 *	−0.34 *	−0.45 **	−0.37 *	0.11	0.11	0.1
II	0.16	0.14	0.3	−0.22	−0.34 *	−0.23	−0.28	−0.38 *	−0.29	−0.03	0.04	0.11
III	0.17	0.24	0.15	−0.35 *	−0.4 *	−0.32 *	−0.41 *	−0.51 **	−0.42 **	0.07	0.14	0.07
IV	0.2	0.19	0.26	−0.27	−0.38 *	−0.3	−0.35 *	−0.45 **	−0.37 *	0.06	0.09	0.11

I. Length; II. Width; III. Third dimension (depth); IV. Volume. * *p* < 0.05; ** *p* < 0.01.

**Table 9:** Duration of menstrual bleeding.

	M ± SD	Min–Max	Me [Q1–Q3]	95% CI	D-C	*p*	Rs
A	5.6 ± 1.5	4–10	5 [5–7]	5.12–6.1	6.12 ^ab^	<0.001	1.00
B	4.7 ± 0.6	4–6	5 [4–5]	4.55–4.9	5.35 ^ac^	<0.001	1.00
C	4.8 ± 0.5	4–6	5 [4–5]	4.62–5	0.76 ^bc^	0.45	−1.00

Friedman ANOVA: χ^2^ = 28.5; df = 2; *p* < 0.001. Post hoc pairwise comparisons were performed using the Durbin–Conover test (D-C). Superscript letters indicate statistically significant pairwise comparisons: ^ab^ = comparison between baseline (A) and 1-month follow-up (B); ^ac^ = comparison between baseline (A) and 3-month follow-up (C); ^bc^ = comparison between 1-month (B) and 3-month follow-up (C).

**Table 10:** Spearman’s rank correlation coefficient (r) between bleeding duration, anthropometric measurements, and procedure duration.

	Before	1 mo	3 mo
Age	−0.18	−0.09	−0.08
Body weight	−0.16	−0.2	−0.35 *
BMI	−0.09	−0.07	−0.24
Procedure duration	−0.12	−0.09	−0.11

* *p* < 0.05;

## Data Availability

The data presented in this study are available on request from the corresponding author.
